# Ameliorative and Antioxidative Potential of *Lactobacillus plantarum*-Fermented Oat (*Avena sativa*) and Fermented Oat Supplemented with Sidr Honey against Streptozotocin-Induced Type 2 Diabetes in Rats

**DOI:** 10.3390/antiox11061122

**Published:** 2022-06-06

**Authors:** Hend F. Alharbi, Raya Algonaiman, Hassan Barakat

**Affiliations:** 1 Department of Food Science and Human Nutrition, College of Agriculture and Veterinary Medicine, Qassim University, Buraydah 51452, Saudi Arabia; hf.alharbi@qu.edu.sa (H.F.A.); 411200162@qu.edu.sa (R.A.); 2 Department of Food Technology, Faculty of Agriculture, Benha University, Moshtohor 13736, Egypt

**Keywords:** fermented oats, *Avena sativa*, honey, *Lactobacillus plantarum*, antidiabetic effects

## Abstract

The ameliorative and antioxidative stress effects of probiotic-enriched fermented oat (FOE) or fermented oat with honey (HFOE) extracts on streptozotocin-induced diabetes in rats were examined. The total phenolic content (TPC) and antioxidant activity (AOA) were increased in FOE and HFOE after 72 h of fermentation, and γ-aminobutyric acid (GABA) reached 7.35 mg 100 g^−1^ in FOE and 8.49 mg 100 g^−1^ in HFOE. The β-glucan levels were slightly decreased to 2.45 g 100 g^−1^ DW in FOE and 2.63 g 100 g^−1^ DW in HFOE. The antidiabetic and hypolipidemic properties of FOE and HFOE were studied in a designed animal model with seven treated groups for 6 weeks. Groups were treated as follows: group 1 (negative group, NR) and group 2 (diabetic rats, DR) were administered 7 mL distilled water orally per day; group 3 (DR + MET) rats were orally administered 50 mg standard drug Metformin kg^−1^ daily; group 4 (DR + FOE1) diabetic rats were orally administered 3.5 mL FOE daily; group 5 (DR + FOE2) rats were orally administered 7 mL FOE daily; group 6 (DR + HFOE1) rats were orally administered 3.5 mL HFOE daily; and group 7 (DR + HFOE2) rats were orally administered 7 mL HFOE daily. The HFOE at the high dose had a synergistic effect, lowering random blood glucose (RBG) and fasting blood glucose (FBG). The hypolipidemic potential of HFOE at the high dose was indicated by significant reductions in triglycerides (TG), total cholesterol (CHO), high- and low-density lipoproteins (HDL and LDL), and very-low-density lipoproteins (VLDL). In addition, 7 mL of HFOE improved liver and kidney function more effectively than other fermented extracts or Metformin. As well as the antioxidant enzyme activity, reduced glutathione (GSH), catalase (CAT), superoxide dismutase (SOD), and malonaldehyde (MDA) were significantly enhanced after the administration of HFOE at 7 mL by 68.6%, 71.5%, 55.69%, and 15.98%, respectively, compared to the DR group. In conclusion, administration of *L. plantarum*-fermented oats supplemented with honey demonstrated antidiabetic effects and a potential approach for controlling glucose levels and lipid profiles, and protecting against oxidative stress.

## 1. Introduction

The effectiveness of common oats (*Avena sativa*) in regulating blood glucose levels has been documented since 1903 [1]. Numerous investigations have reported oats’ therapeutic effects on diabetes and multiple other health issues, such as cardiovascular disease, hypertension, and dyslipidemia [2,3]. Soluble dietary fiber, namely β-glucan, plays a significant role in reducing cholesterol, glucose, and insulin levels [4,5,6]. The potent antioxidant capacity of oats, mainly derived from the oats’ unique polyphenols, known as avenanthramides, also delivers multiple health-promoting effects, such as antiatherosclerosis and anti-inflammatory activities [7,8].

Oat fermentation has been shown to significantly increase the antioxidant capacity much more so than non-fermented oat products [9,10], due to the changes and release of multiple nutrients during fermentation [5]. These changes result from the microbial metabolic activities that mainly break down carbohydrate fractions into several end products, such as acids. The presence of multiple end products and the release of several phytochemicals deliver various health-promoting properties, such as the prevention of cardiovascular disease [11], hypertension [12,13,14,15], obesity [16,17], and osteoporosis [18]. In addition, a variety of fermented foods have shown antidiabetic effects both in vitro and in vivo [6] using a wide range of different microorganisms as starter cultures. The well-known *Lactobacillus* species, such as *L. fermentum*, *L. acidophilus*, and *L. plantarum*, have been widely used as probiotics in the food industry [19]. Various strains have been reported to exert health-promoting activities, such as immunomodulation and antipathogenic activities, and have high cholesterol-lowering efficiencies [20].

Furthermore, a recent in vitro investigation [21] reported that *L. plantarum* promotes antidiabetic effects. A similar strain was also used in vivo to investigate its antidiabetic effects [22]. Their results showed a reduction in blood glucose levels, and the authors indicated that *L. plantarum* is a potential therapeutic agent for the management of diabetes. Moreover, a recent investigation has shown that the fermentation of oats with the addition of honey delivers even more desirable results [11], such as maintaining the concentrations of β-glucan at optimum levels. β-glucan is a selective substrate of *Lactobacillus* [23], and thus, the addition of honey provides an energy source for bacterial growth.

The development of a new dietary intervention for the management of diabetes is highly urgent. The prevalence of diabetes has increased dramatically in recent years. Statistical results showed that more than 6 million deaths were caused directly by diabetes in 2021 [24,25]. In Saudi Arabia, diabetes is the 10th highest cause of death [26]. The prevalence of new cases is also growing day by day. About 50% of the Saudi population are predicted to be diabetic by 2030 [19,27]. Therefore, the prevention of diabetes and seeking new dietary interventions is much needed. This study investigates the antidiabetic efficiency in terms of the ameliorative and antioxidative stress potential of *L. plantarum*-fermented oat extract (FOE) and fermented oat extract supplemented with honey (HFOE) on several biomarkers in animal diabetic models. To the best of our knowledge, the antidiabetic potential of HFOE using the *L. plantarum* strain in the form of a functional beverage has not been studied yet.

## 2. Materials and Methods

### 2.1. Ingredients, Chemicals, and Strain

Whole oat grains were purchased from the local store of Buraydah, Saudi Arabia (manufactured by Federal Oats Mills, 13400, Butterworth, Malaysia). The nutritional value of oats per 100 g consists of 370 kcal, 12.1 g protein, 8.4 g fat, 56.1 g carbohydrates, 1.0 g sugar, 10.8 g dietary fiber, 4.0 mg iron, 110.0 mg magnesium, 3.0 mg zinc, 52.0 µg folic acid, 1.0 mg, 0.4 mg, and 0.4 µg of vitamin A, B1, and B12, respectively. Sidr honey was obtained from the local market of Riyadh, Saudi Arabia.

A strain of *Lactobacillus plantarum* (NRRL B-59151) was generously provided by the USDA Agricultural Research Service (ARS) Culture Collection (Peoria, IL, USA), and MRS broth was purchased from Condalab (Madrid, Spain). Streptozotocin (STZ), >97% purity, was purchased from Alfa Aesar, Thermo Fisher Scientific (Kandel, Germany), and Metformin in pure form was purchased from Sigma-Aldrich (Saint Louis, MO, USA). A mixed-linkage β-glucan kit was purchased from Megazyme International (Bray, Ireland), and γ-aminobutyric acid (GABA) analytical standard was purchased from Sigma-Aldrich (Saint Louis, MO, USA).

### 2.2. Preparation of Fermented Oat (FOE) and Honey-Supplemented Fermented Oat (HFOE) Extracts

The preparation of the fermented extracts was performed following the method of Chen et al. [11], with slight modifications. Briefly, a suspension of 2% Sidr honey in distilled water was pasteurized at 80 °C for 10 min. The suspension was then added to sterilized whole oat flour to yield a concentration of 10% oats (*w*/*v*). After cooling down to room temperature, the starter culture of *L. plantarum* was inoculated at 1% (*v*/*v*). The mixture was then fermented at 37 °C for 24, 48, and 72 h in a microbiological incubator. A fermented oat mixture without incorporating honey was prepared following the same method. After fermentation, samples were centrifuged at 12,000× *g* for 15 min at 4 °C. The collected supernatants were stored at 4–8 °C for 7 days. These extracts were orally administered to the rats, and fresh-made extracts were prepared each week.

### 2.3. Enumeration of L. plantarum B-59151 Viable Count

The viable count of *L. plantarum* B-59151 in FO and HFO was enumerated following the standard plate count method according to Vinderola and Reinheimer [28]. Firstly, 10 mL of each sample was suspended in 90 mL of sterile peptone water (Merck, 0.1%), after which it was serially diluted. Aliquots of 1 mL of appropriate dilutions were inoculated on sterile plates of MRS agar. MRS agar-inoculated plates were incubated at 37 °C for 48–72 h in anaerobic jars (2.5 L) with a GasPak system (GasPak System-Oxoid, Basingstoke, Hampshire, UK) according to standard methods [28]. Data are expressed as the logarithm of colony forming units per mL (Log_10_ CFU mL^−1^).

### 2.4. Determination of Total Phenolic Content (TPC) in FOE and HFOE

The TPC of FOE and HFOE was determined using Folin–Ciocalteu reagent, according to Yawadio Nsimba et al. [29]. Briefly, in Eppendorf tubes, 150 µL of the sample was mixed with 300 µL of Folin–Ciocalteu reagent for 5 min. Then, 300 µL of an alkali solution (7.5% sodium carbonate solution, Na_2_CO_3_) was added. The mixture was incubated in the dark for 60 min at 23 °C, then centrifuged at 10,000× *g* for 10 min at 4 °C, and 200 µL of supernatant from each Eppendorf was transferred to a new 96-well plate; the absorbance was then measured at 765 nm using a microplate reader (BioTek, Winooski, VT, USA). Measurements were compared against the standard curve of gallic acid (GA) solution (R^2^ = 0.99), and TPC content was expressed as milligrams of Gallic acid equivalents (GAE) per gram of dry weight (DW) (mg of GAE g^−1^ DW).

### 2.5. Determination of Total Antioxidant Capacity (TAC) of FOE and HFOE

The TAC of FOE and HFOE was measured spectrophotometrically based on the bleaching of DPPH radicals in purple solution according to Yawadio Nsimba et al. [29]. Briefly, 600 µL of DPPH solution was added to 100 µL of sample in Eppendorf tubes. After incubation in the dark for 60 min at 25 °C, tubes were centrifuged at 10,000× *g* for 5 min at 4 °C, after which, 200 µL of supernatant from each Eppendorf was transferred to a new 96-well plate; the absorbance was then measured at 517 nm using a microplate reader (BioTek, Winooski, VT, USA). The DPPH radical scavenging activity percentage was calculated based on the plotted Trolox calibration curve. The antiradical activity was expressed as micromoles of Trolox equivalents (TE) per gram of dry weight (µmol TE g^−1^ DW). The ABTS radical scavenging activity of FOE and HFOE against ABTS radicals was tested by the adapted method of Lu et al. [30].

### 2.6. Determination of the γ-Aminobutyric Acid (GABA) Content

The content of GABA was evaluated spectrophotometrically according to Yeap et al. [31]. Briefly, 0.5 mL of extract was mixed with 0.5 mL of borate buffer, 0.5 mL of 6% phenol reagent, and 1.5 mL of 6% sodium hypochlorite (NaClO); the mixture was then boiled at 100 °C for 10 min and immediately cooled down in a cooling bath. The absorbance was then measured at 630 nm. A GABA standard was used to prepare a standard curve, and the results were expressed as mg 100 g^−1^ DW.

### 2.7. Determination of the β-Glucan Content

The β-glucan content in the fermented oat mixtures was quantified using an enzymatic mixed-linkage assay kit (Megazyme International Co., Wicklow, Ireland). According to the manufacturer’s protocol. All measurements were performed twice and in duplicate, following the method of a previous study [11]. β-glucan content was expressed as g 100 g^−1^ DW.

### 2.8. Animals and Experimental Design

This study used Wistar rats (56 adult males) weighing between 170 and 190 g. Under standard laboratory conditions, the animals were housed in air-conditioned polypropylene cages and kept at 24 ± 1 °C, 40–45% relative humidity, on a 12 h light/dark cycle, and were fed a commercial standard pellet diet and given water ad libitum [32]. All experiments were approved by the Committee of Research Ethics (Institutional Review Board, IRB) of Qassim University, Saudi Arabia (Approval No. 21-13-19 on 21 March 2022).

After ten days of acclimatization, rats were randomly divided into 7 groups (8 rats/group). The first group, normal rats (NR), received an intraperitoneal injection of saline solution and 5 mL distilled water orally per day. The rest of the groups were fasted overnight and received a single intraperitoneal injection of freshly prepared solution of STZ in 0.1 M citrate buffer (pH = 4.5) at a dose of 45 mg kg^−1^ BW, to induce diabetes. The confirmation of diabetes was assessed by monitoring the fasting blood glucose (FBG) levels after 48 h of STZ injection using a glucometer (Accu-Check, Roche, Germany). Experimental rats with an FBG level > 200 mg dL^−1^ were considered diabetic, and were included in the study. Animals were randomized based on their body mass and random blood glucose (RBG) levels, and divided into 6 groups, as shown in Table 1. Metformin was chosen as a reference drug as it is the first-line medication for treating type 2 diabetes [33].

At the end of the 6th week, 12 h-fasted animals were anesthetized with a mixture of alcohol, chloroform, and ether (1:2:3). Collected blood samples were subjected to serum separation by centrifugation at 4000× *g* for 30 min under cooling to attain serum used for various biochemical parameters. The biochemical parameters were determined using suitable kits and a blood chemistry analyzer (HumaLyzer 4000, Human Gesellschaft für Biochemica und Diagnostica mbH, Wiesbaden, Germany).

#### 2.8.1. Determination of Fasting Blood Glucose Level (FBG), Lipid Profile, and Liver and Kidney Functions

The GOD–PAP method was used to determine FBG (mg dL^−1^) using an enzymatic colorimetric test kit. Triglycerides (TG, mg dL^−1^) and total cholesterol (CHO, mg dL^−1^) were determined using an enzymatic colorimetric test kit and the GPO–PAP method, respectively. High-density lipoproteins (HDL, mg dL^−1^) were determined using an enzymatic colorimetric direct homogenous test kit following manufacturer protocols. According to Friedewald et al. [34], low-density lipoproteins (LDL, mg dL^−1^) and very-low-density lipoproteins (VLDL, mg dL^−1^) were calculated mathematically using indicated equations. The liver function biomarkers, such as alanine aminotransferase (ALT, UL^−1^), aspartate aminotransferase (AST, UL^−1^), alkaline phosphatase (ALP, UL^−1^), and total bilirubin (T. Bili, mg dL^−1^) in blood serum were examined using an alanine aminotransferase kit (EC 2.6.1.2), aspartate aminotransferase kit (EC 2.6.1.1), and an alkaline phosphatase kit (2.6.1.4). Kidney function biomarkers, such as total protein (T. Protein, g dL^−1^), albumin (g dL^−1^), creatinine (mg dL^−1^), and urea (mg dL^−1^) concentrations were respectively determined using photometric and colorimetric test kits applying the Biuret method, photometric and colorimetric test kits applying the BCG method, photometric and colorimetric test kits, and a fully enzymatic test kit applying the GLDH method, according to the instructions of the manufacturer. Globulin concentrations (g dL^−1^) were calculated by subtracting albumin from T. Protein concentrations. Blood urea nitrogen (BUN, mg dL^−1^) was calculated by multiplying urea concentration by 0.47. All biochemical examination kits were purchased from Human Co., Wiesbaden, Germany. The atherogenic index (AI) was calculated according to Nwagha et al. [35].

#### 2.8.2. Oxidative Stress Biomarkers

Reduced glutathione (GSH, µg dL^−1^) was estimated using a GSH colorimetric assay kit (E-BC-K030-S, Elabscience, Houston, TX, USA) according to the method described by Beutler et al. [36]. Lipid peroxidation was assessed using a malondialdehyde (MDA, nmol mL^−1^) colorimetric assay kit (E-BC-K025-S, Elabscience, Houston, TX, USA) measuring TBARS, and expressed in terms of MDA content according to Ohkawa et al. [37]. MDA, a byproduct of fatty acid peroxidation, reacts with thiobarbituric acid to form a colored complex (TBA). The absorbance of the supernatant was measured at 532 nm and converted to nmol mL^−1^. Superoxide dismutase (SOD, U L^−1^) activity was determined using a SOD activity assay kit (E-BC-K022-S, Elabscience, Houston, TX, USA) according to Giannopolitis and Ries [38]. At 550 nm, the color reaction was measured and expressed as U L^−1^. The activity of catalase (CAT, U L^−1^) was measured using a CAT activity assay kit (E-BC-K031-S, Elabscience, Houston, TX, USA) and the Aebi method [39]. A blood chemistry analyzer was used to determine all oxidative stress markers (HumaLyzer 4000, Human Gesellschaft für Biochemica und Diagnostica mbH, Wiesbaden, Germany).

### 2.9. Statistical Analysis

The statistical analysis was carried out by applying a one-way ANOVA for phytochemicals and antioxidant capacity, γ-aminobutyric acid content, β-glucan content, lipid profile parameters, liver and kidney functions, and antioxidant biomarker data, and a two-way ANOVA for *L. plantarum* viable count, and pH and RBG data using SPSS (Ver. 22.0 for Windows). Experimental results are expressed as mean ± standard error. Applying Tukey’s test, multiple comparisons were carried out, and the significance level was set at <0.05. Data were treated as a complete randomization design according to Steel et al. [40].

## 3. Results

### 3.1. Survival of Lactobacillus plantarum and Related pH Value in FOE and HFOE

**Figure 1:**
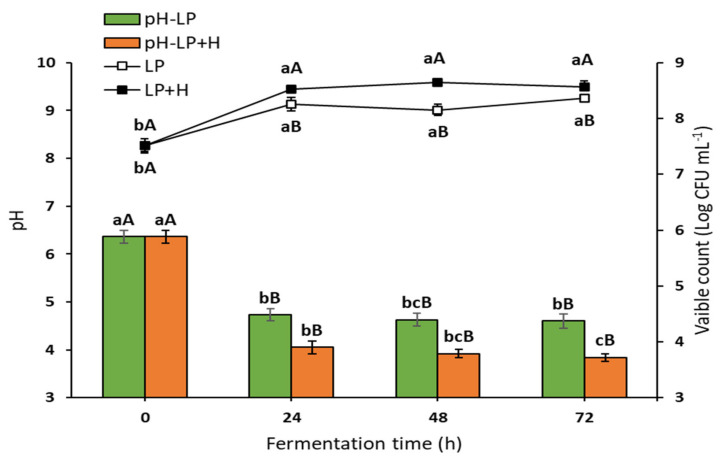
Viability of *L. plantarum* (Log CFU mL^−1^) and pH value during fermentation (mean ± SE), *n* = 3. LP, *L. plantarum* count in plain media; LP + H, *L. plantarum* count in media with 2% Sidr honey; pH-LP, pH value of basic media during *L. plantarum* fermentation; pH-LP + H, pH value of media with honey during *L. plantarum* fermentation. ^a–c^ Bars or lines during the fermentation period not sharing similar letters are significantly different (*p* > 0.05). ^A,B^ Bars or lines of each treatment not sharing similar letters during the fermentation time are significantly different (*p* > 0.05).

The current investigation used sterilized oat flour, obtained from whole oat grains, in water (1:9, *w*/*v*) as the fermentation media for *L. plantarum* (FOE), and (1:9, *w*/*v*) with 2% Sidr honey (HFOE). Immediately, *L. fermentum* was inoculated into both media in the range of 7.45–7.51 (CFU mL^−1^) using a freshly prepared starter culture. The growth profile, as shown in Figure 1, demonstrated that there was a significant increase in the viable count during the first 24 h (8.25 log CFU mL^−1^ for FOE and 8.52 log CFU mL^−1^ for HFOE) as compared to 0 h, with a slight decrease after 48 h (8.16 log CFU mL^−1^ for FOE) and a slight increase after 48 h (8.65 log CFU mL^−1^ for HFOE). Indeed, the viable count remained relatively stable during this period, confirming the steady growth of *L. plantarum*. The viable cell count was 8.36 and 8.57 log CFU mL^−1^ in FOE and HFOE, respectively, at maximum fermentation time (72 h). This increase in cell growth was accompanied with a significant decrease in pH values from 6.36 to 4.73 after 24 h, 4.63 after 48 h, and a further reduction to 4.60 at 72 h for FOE, and from 6.36 to 4.05 after 24 h, 3.92 after 48 h, and a further decrease to 3.84 at 72 h for HFOE. Interestingly, adding honey to the fermentation media accelerated the growth, reflected in the cell count and pH level.

### 3.2. Phytochemicals and Antioxidant Capacity of FOE and HFOE

The TPC and relative antioxidant activities using DPPH and ABTS assays of FOE and HFOE were investigated; data are presented in Table 2. TPC was significantly increased with the long fermentation time in both extracts. Interestingly, HFOE showed a significant increase in TPC, much more than FOE. Accordingly, the antioxidant capacity increased with the fermentation time in all subjected extracts; the highest values were recorded in HFOE after 72 h for both DPPH and ABTS radicals.

### 3.3. Gamma-Aminobutyric Acid and β-Glucan Contents during Fermentation

The gamma-aminobutyric acid (GABA) and β-glucan contents in FOE and HFOE were screened (Table 3). The GABA content increased significantly after 48 h to 6.10 ± 0.52 mg 100 g^−1^ and 6.54 ± 0.21 mg 100 g^−1^, in FOE and HFOE, respectively. After 72 h, GABA showed the most significant increase (7.35 ± 0.40 mg 100 g^−1^ in FOE and 8.49 ± 1.13 mg 100 g^−1^ in HFOE; *p* < 0.05). Furthermore, monitoring β-glucan as a bioactive component in oats during fermentation was of interest in the present study. There were no significant changes in the β-glucan content during the first 48 h of fermentation in FOE and HFOE. A slight, nonsignificant decrease in FOE and HFOE after 48 h was observed (2.56 ± 0.03 g 100 g^−1^ DW and 2.67 ± 0.01 g 100 g^−1^ DW, respectively). After 72 h, β-glucan content was 2.45 ± 0.06 g 100 g^−1^ DW, which differed significantly compared to its content in FOE during the period of 0–48 h. On the contrary, no significant difference was found in β-glucan content during fermentation of HFOE.

### 3.4. The Hypoglycemic Efficiency of FOE and HFOE

The hypoglycemic efficiency of FOE and HFOE at 3.5 mL and 7 mL, and Metformin at 50 mg kg^−1^, on STZ-induced diabetic rats was monitored Table 4. STZ injection affected the rats’ RBG directly during the first week. The administration of all fermented oat extracts showed significant improvement in the RBG recorded in the third and the sixth weeks compared to the DR group, as shown in Table 4. The most efficient treatment for improving the rats’ RBG levels was the HFOE2. In addition, FBG measurement confirmed that FOE and HFOE impressively attenuated the levels close to normal rats (NR group). Interestingly, FOE2 and HFOE2 significantly attenuated the serum glucose levels, and there were no significant differences compared to the NR or Metformin groups confirming the potential hypoglycemic efficiency.

### 3.5. The Hypolipidemic Efficiency of FOE and HFOE

The hypolipidemic efficiency of FOE and HFOE at 3.5 mL and 7 mL on the STZ-induced diabetic rats was determined and shown in Table 5. A significant increase in TG, CHO, LDL, and VLDL levels, while a significant decrease in HDL levels in the positive control group (DR) compared to the NR group, was noted (*p* < 0.05). The administration of all fermented oat extracts significantly attenuated the TG, CHO, LDL-CHO, and VLDL-CHO levels compared to the DR group. The high doses, FOE2 and HFOE2, significantly increased the HDL-CHO and decreased VLDL-CHO levels. Interestingly, the HFOE2 showed the most potent effects in improving serum lipids. Moreover, the AI was increased significantly after STZ injection (DR group) compared to the NR group, and the most efficient extracts in attenuating the atherogenicity complication were the high doses of both extracts (FOE2 and HFOE2), which present a superior effect to both the low doses and the Metformin treatment.

### 3.6. The Liver Functions

STZ injection substantially raised serum ALT, AST, and ALP enzyme levels in DR rats compared to the NR group (*p* < 0.05); in addition, the T. Bili levels were significantly increased in the DR group, as shown in Table 6. Administration of FOE or HFOE promoted hepatoprotective effects. The high doses exhibited much more effective results than using lower doses; FOE2 and HFOE2 treatments substantially reduced ALT, AST, and ALP to normal levels, even more effectively than Metformin.

### 3.7. The Kidney Functions

The nephroprotective efficiency of FOE1, FOE2, HFOE1, HFOE2, and Metformin at a dose of 50 mg kg^−1^ in the STZ-induced diabetic rats was investigated (Table 7). STZ injection substantially raised serum creatinine, urea, and BUN levels in DR rats compared to normal rats (DR). Contrarily, T. Protein, albumin, and globulin levels were significantly decreased. Treatments with FOE1, FOE2, HFOE1, and HFOE2 considerably improved the creatinine, urea, and BUN concentrations. Additionally, the T. Protein, albumin, and globulin levels were raised to normal levels, as seen in Table 7. The HFOE2 showed the most significant improvement in these levels compared to normal rats, even better than Metformin treatment.

### 3.8. Antioxidant Biomarkers

**Figure 2:**
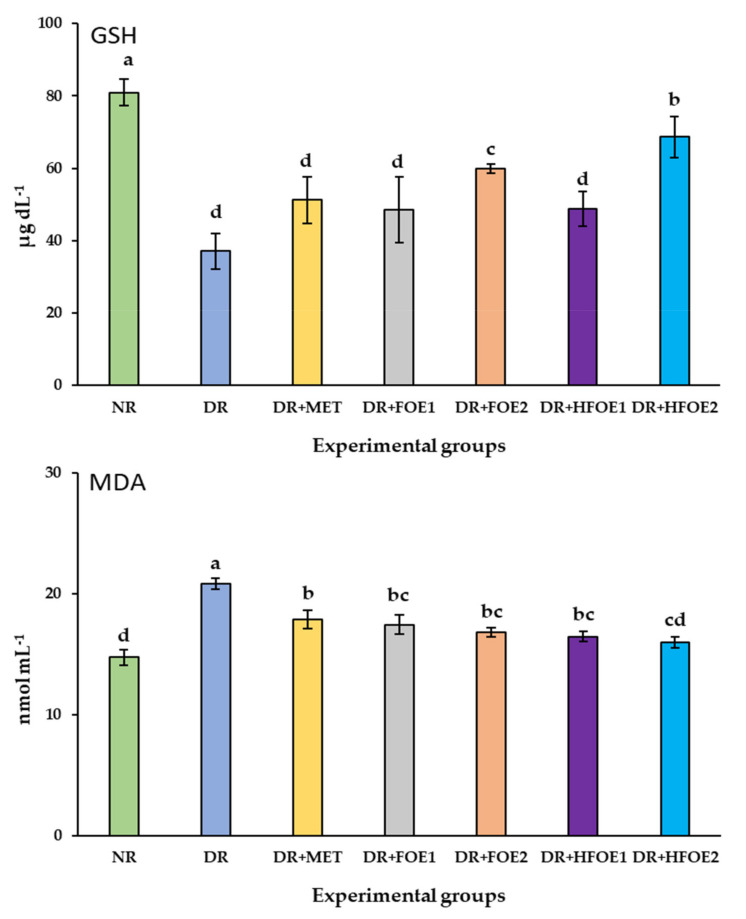

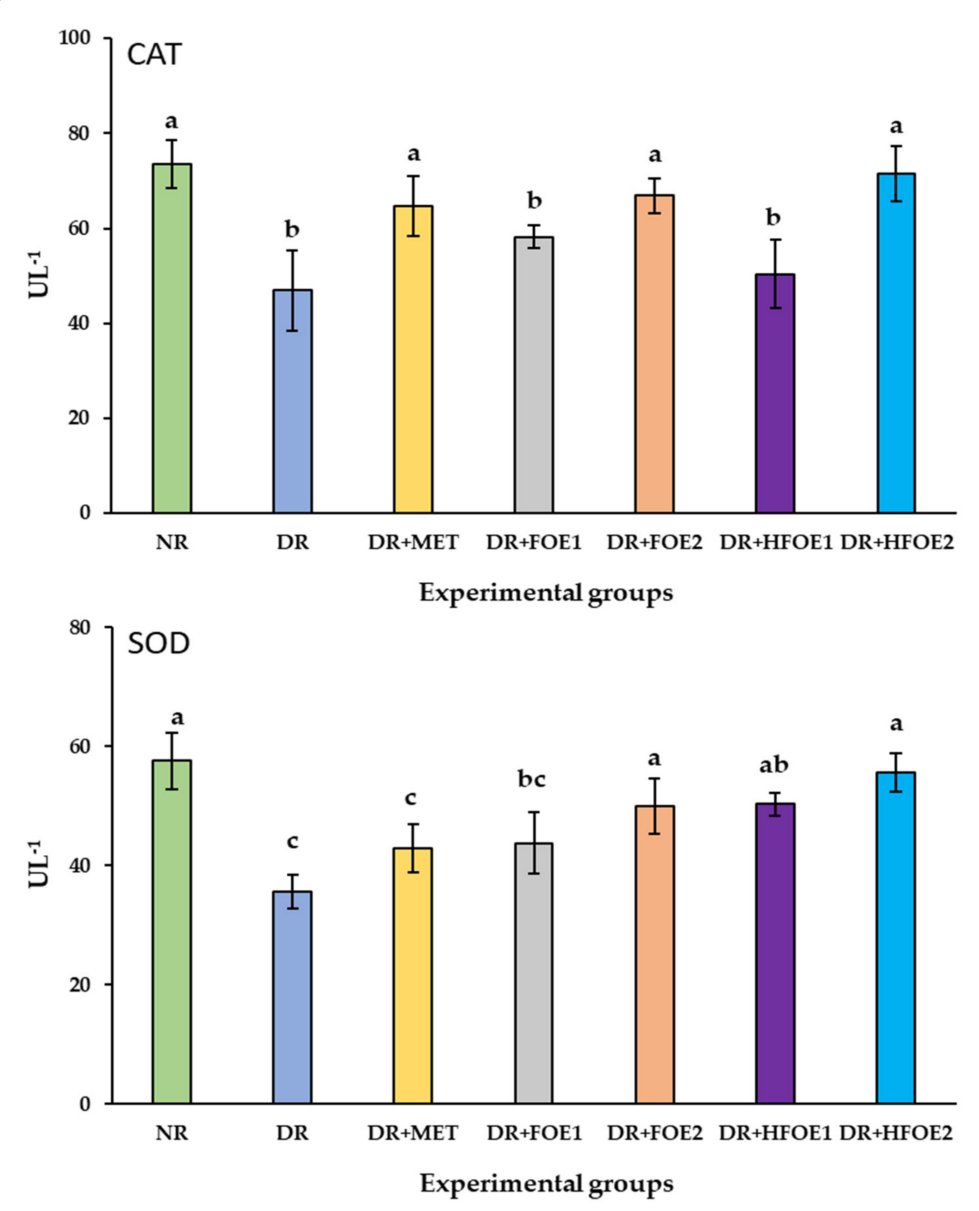
Effect of orally administrated FOE and HFOE on antioxidant biomarkers in streptozotocin-induced diabetes in rats (mean ± SE), *n* = 8. Experimental groups, see materials and methods; Section 2.8. GSH, reduced glutathione; MDA, malondialdehyde; SOD, superoxide dismutase; CAT, catalase. ^a–d^ Bars not sharing similar letters are significantly different (*p* > 0.05).

The effect of FOE1, FOE2, HFOE1, HFOE2, and Metformin on antioxidant biomarkers was evaluated. As shown in Figure 2, STZ injection decreased GSH, CAT, and SOD enzyme levels, while it increased MDA levels in DR rats’ blood serum compared to normal rats (NR) (*p* < 0.05). The administration of all treatments resulted in a considerable increase in the activity of antioxidant enzymes GSH, CAT, and SOD, and a significant decrease in MDA levels. Treatments with all fermented extracts improved the antioxidant biomarkers, and their levels were significantly improved in a dose-dependent manner. Interestingly, HFOE2 dramatically improved the enzymatic defense system compared to all DR rats.

## 4. Discussion

In recent years, cereal fermentations have been significantly demonstrated as promising functional products for improving or enhancing a foods’ nutritional quality. Several health-promoting effects of fermented products have been reported [10]. Lactic acid bacteria were reported as the most commonly used starter cultures in the food industry [21,22,41]. In the current work, aqueous extracts of *L. plantarum*-fermented oat and fermented oat supplemented with 2% Sidr honey were beneficial for managing diabetes complications in STZ-induced diabetic rats. The aqueous extracts were further subjected to multiple investigations to assess the changes in the nutritional quality.

Our results show that fermentation by *L. plantarum* significantly altered the composition of the oats’ aqueous extracts. There was a significant increase in total phenolic compounds and the related antioxidant capacity in a time- and type-dependent manner; the longer the fermentation time, the higher the release of total phenolic compounds. These results agree with multiple studies [11,42]; the long fermentation time (72 h) resulted in the most significant increase in total phenolic compounds. Biologically, phenolic compounds are considered as health-promoting agents [43,44,45,46]. Cereal fermentation can modify the presence of these compounds due to enzymatic activities derived from both the grains and the microbes [23]; enzymes such as glycosidase, hydrolase, esterase, and β-glucosidase produced by different types of bacterial strains can improve the availability of the grains’ phenolic compounds [11]. In addition, fermentation could increase the presence of other bioactive compounds, such as GABA [47], which is a non-protein amino acid that works as a neurotransmitter in the mammalian brain [48]; it has been demonstrated to play several roles in health-promoting activities, such as antihypertension and antidiabetic activities [49]. In the present study, we evaluated the effect of fermentation on the release of GABA; once again, the results were consistently time- and type-dependent. After 72 h of fermentation, we found a significant increase in GABA content by 78.3% and 92% in both FOE and HFOE, respectively. Previous studies reported similar results in fermented oats [50,51].

Interestingly, our results show that the GABA content in HFOE was much higher than that in FOE; such results could be attributed to the prebiotic effects of honey [52]. It was also indicated that GABA production could be affected by multiple factors, including fermentation time and microbe type [47], which supports our findings. The longer fermentation time (72 h) and the use of the *L. plantarum* strain significantly affected GABA production. The antidiabetic activities of GABA are attributed to its effects on promoting insulin secretion [49]. In the present in vivo investigation, the administration of FOE and HFOE, which were subjected to 72 h of fermentation, showed significant results in improving RBG and FBG. However, further investigation conducting complete GC–MS/MS and HPLC analyses is highly recommended to gain a better overview of the performance of such components during fermentation.

Moreover, oats’ β-glucans are documented as a potent antidiabetic agent [53,54,55]. However, previous studies indicated that β-glucan levels could be reduced during harsh processing [56]. In the interest of the present study, there was a slight decrease in the β-glucan content by 6.5% and 3.3% in FOE and HFOE, respectively, after 72 h of fermentation. Interestingly, a significant difference between both extracts in maintaining the content of β-glucan was observed, which is strongly attributed to the incorporation of honey [11]. Chen et al. [11] support this notion; the incorporation of honey in fermented oat-based beverages showed no significant decrease in the β-glucan content.

On the other hand, prolonged hyperglycemia is a primary cause of most diabetic complications. Indeed, chronic hyperglycemia leads to various metabolic impairments [57]. In the present study, after 6 weeks of oral administration of *L. plantarum*-fermented oat extracts, our results show significant hypoglycemic effects in STZ-induced diabetic rats in a dose- and type-dependent manner. The incorporation of honey in the fermented oat extract (HFOE2) strongly reduced the RBG and FBG levels compared to FOE1, FOE2, and HFOE1. These results could be related to the prebiotic properties of honey [52]. Moreover, honey was demonstrated to contain tremendous amounts of good-quality phytochemicals, such as flavonoids and phenolic acids, which could significantly improve glucose levels [58,59]. In addition, honey has antimicrobial, anti-inflammatory, and wound-healing properties that have been used in ancient and modern medical practice [60]. These beneficial effects of honey may partly explain the improvements in glycemic control of diabetic animals in this study [1,4]. Therefore, honey is an appropriate ingredient in functional products [61], which could support the functionality of prepared extracts.

Furthermore, imbalances in serum lipids were observed due to the injection of STZ, indicating impairment in fat metabolism due to diabetes complications [4]. The administration of FOE1, FOE2, HFOE1, and HFOE2 significantly enhanced the drastic changes in serum lipids. Interestingly, HFOE2 showed much more significant effects in improving TG, CHO, and LDL-cholesterol levels, which could also be related to the strong presence of phenols and β-glucan [11,61]. Oats were documented in several studies to exert therapeutic effects, improving blood glucose levels and CHO levels [1,62]. In the present study, the desirable results in strengthening hyperglycemia and serum lipid levels demonstrate that oat fermentation supplemented with excellent prebiotics such as honey may increase the potential therapeutic qualities of oats in the management of diabetes and the diabetes-related complications.

Furthermore, significant differences in liver and kidney diagnostic markers were observed in the treated groups compared to the positive control group. The significant increase in ALT, AST, ALP, and T. Bili enzyme activities in the positive control group due to the STZ injection represents normal deterioration associated with diabetic liver injury [44,46]. The intervention of all subjected extracts resulted in significant improvements in balancing the liver enzyme levels in a dose- and type-dependent manner. Bioactive compounds derived from the activity of probiotic strains during fermentation could explain these desirable hepatoprotective effects [61,63]. Probiotic supplementation could prevent hepatic steatosis by improving and treating metabolic disorders, balancing the lipid profile, and suppressing the inflammatory state [63,64,65]. Consistently, imbalances in kidney enzymes were observed due to significant hyperglycemia. The higher the blood glucose levels, the more damage to the kidney filtering units, resulting in kidney failure [66,67]. This has led to diabetes becoming one of the leading causes of end-stage kidney disease, also known as diabetic nephropathy [66]. In our investigation, the recovery of all biomarkers of kidney functions in the diabetic rats was observed after the intervention of all fermented oat extracts in a dose- and type-dependent manner.

Interestingly enough, an increase in T. Protein and albumin levels, concomitant with decreases in urea and BUN levels, were highly significant after administering HFOE2 compared to all other groups. In a previous study [66], kidney function parameters were recovered to a normal state after the intervention of oats [68]. Oat extract was related to a reduction in the liver’s glucose production and increased insulin secretion, which is associated with improved kidney functions [64,67]. On the other hand, the injection of STZ also resulted in significant imbalances in oxidative stress markers. A decrease in serum GSH, SOD, and CAT levels, and an increase in MDA levels, was observed. In hyperglycemia, various mechanisms generate free radicals, leading to the oxidation of lipids, proteins, and DNA, resulting in a significant state of oxidative stress [69]. The increase in free radicals reduces the production of biological antioxidative enzymes such as CAT, SOD, and GSH [70], making the tissues more vulnerable to oxidative stress [71]. The intervention of all fermented oat extracts in the present study recovered the altered activity of antioxidant enzymes in diabetic rats. Consistently, we found that HFOE2 was more effective than the other subjected extracts and even more effective than Metformin treatment at preventing oxidative stress. These effects are strongly related to a decrease in lipid peroxidation [71], which could be attributed to the combination of the antioxidant activities of oats, honey, and *L. plantarum* metabolites [58,66,71]. Therefore, our results support the hypothesis that a good-quality cereal-based symbiotic product possesses potent antidiabetic activities with antioxidative stress potential.

## 5. Conclusions

In this study, *L. plantarum*-fermented oat or fermented oat combined with Sidr honey exhibited potential antidiabetic effects, including ameliorative and antioxidative stress effects. We innovatively investigated the antidiabetic and antioxidative potential of FOE and HFOE in the form of aqueous extracts. Our results show a significant enhancement in the total phenolic compounds, the antioxidant capacity, and the gamma-aminobutyric acid in a type- and time-dependent manner. Fermentation up to 72 h and the incorporation of honey showed much more desirable effects. Moreover, honey proved to be a good prebiotic source, which was reflected in maintaining the oats’ β-glucan levels. Furthermore, the oral administration of HFOE for 6 weeks effectively improved diabetic complications in diabetic rats by improving blood glucose levels, lipidemia, liver and kidney functions, and oxidative stress markers. This study demonstrates that incorporating honey in *L. plantarum*-fermented oats has high potential as a symbiotic product for the management of diabetes. Future strategies for producing functional supplements based on these data are highly recommended.

## Figures and Tables

**Table 1:** Experimental design of streptozotocin-induced diabetic rats treated with FOE and HFOE daily for 6 weeks.

Group	Experimental Treatment
NR	Normal rats
DR	Untreated diabetic rats + distilled water (7 mL)
DR + MET	Diabetic rats + Metformin (50 mg kg^−1^)
DR + FOE1	Diabetic rats + FOE (3.5 mL)
DR + FOE2	Diabetic rats + FOE (7 mL)
DR + HFOE1	Diabetic rats + HFOE (3.5 mL)
DR + HFOE2	Diabetic rats + HFOE (7 mL)

FOE, fermented oat extract; HFOE, honey-supplemented fermented oat extract. All rats received a standard rodent diet during the entire experiment.

**Table 2:** Total phenolic content (TPC) and potential antioxidant activities in FOE and HFOE during fermentation up to 72 h (mean ± SE), *n* = 8.

Fermentation Time		Item		
		TPC (mg GAE g^−1^)	DPPH (µmol of TE g^−1^)	ABTS (µmol of TE g^−1^)
FOE	0 h	0.70 ± 0.08 ^b^	2.14 ± 0.09 ^b^	3.47 ± 0.29 ^c^
	24 h	0.74 ± 0.07 ^b^	2.22 ± 0.11 ^b^	3.89 ± 0.13 ^c^
	48 h	0.94 ± 0.15 ^ab^	3.19 ± 0.12 ^ab^	4.58 ± 0.31 ^b^
	72 h	1.04 ± 0.08 ^a^	3.56 ± 0.21 ^a^	5.27 ± 0.14 ^a^
HFOE	0 h	1.41 ± 0.13 ^b^	5.12 ± 0.15 ^c^	7.68 ± 0.31 ^d^
	24 h	1.48 ± 0.12 ^ab^	6.01 ± 0.22 ^b^	9.14 ± 0.47 ^c^
	48 h	1.52 ± 0.06 ^a^	6.47 ± 0.41 ^b^	9.71 ± 0.27 ^b^
	72 h	1.64 ± 0.14 ^a^	7.15 ± 0.24 ^a^	11.73 ± 0.16 ^a^

^a–d^ No significant difference (*p* > 0.05) between any two means within the same column that have the same superscripted letters.

**Table 3:** The γ-aminobutyric acid and β-glucan content in FOE and HFOE during fermentation up to 72 h (mean ± SE), *n* = 8.

Fermentation Time		Item	
		GABA * (mg 100 g^−1^)	β-Glucan (g 100 g^−1^)
FOE	0 h	4.12 ± 0.14 ^b^	2.62 ± 0.02 ^a^
	24 h	4.77 ± 0.17 ^b^	2.60 ± 0.01 ^a^
	48 h	6.10 ± 0.52 ^ab^	2.56 ± 0.03 ^a^
	72 h	7.35 ± 0.40 ^a^	2.45 ± 0.06 ^b^
HFOE	0 h	4.42 ± 1.02 ^c^	2.72 ± 0.14 ^a^
	24 h	4.78 ± 0.49 ^c^	2.69 ± 0.04 ^a^
	48 h	6.54 ± 0.21 ^b^	2.67 ± 0.01 ^a^
	72 h	8.49 ± 1.13 ^a^	2.63 ± 0.03 ^a^

* γ-aminobutyric acid. ^a–c^ No significant difference (*p* > 0.05) between any two means within the same column that have the same superscripted letters.

**Table 4:** Effect of orally administrated FOE and HFOE on RBG and FBG (mg dL^−1^) in STZ-induced diabetes in rats (mean ± SE), *n* = 8.

Groups *	RBG			FBG
	Week 0	Week 3	Week 6	
NR	113.83 ± 3.82 ^dA^	114.33 ± 3.06 ^dA^	110.67 ± 2.55 ^dA^	87.16 ± 4.44 ^b^
DR	254.17 ± 33.65 ^aA^	286.33 ± 36.6 ^aA^	299.50 ± 29.11 ^aA^	156.27 ± 13.34 ^a^
DR + MET	284.33 ± 24.90 ^abA^	238.67 ± 40.01 ^bcB^	201.50 ± 28.49 ^bcC^	89.39 ± 3.25 ^b^
DR + FOE1	252.5 ± 36.93 ^bcA^	237.5 ± 20.92 ^bcA^	218.67 ± 4.66 ^bcB^	102.77 ± 10.17 ^b^
DR + FOE2	308.5 ± 22.13 ^abA^	244.83 ± 28.82 ^abB^	232.50 ± 15.24 ^bB^	82.03 ± 7.55 ^b^
DR + HFOE1	276.67 ± 37.38 ^bcA^	254.33 ± 29.58 ^abB^	241.50 ± 25.39 ^bB^	94.04 ± 10.36 ^b^
DR + HFOE2	320.83 ± 39.36 ^aA^	225.17 ± 18.35 ^bcB^	211.50 ± 32.66 ^bcC^	86.48 ± 11.13 ^b^

* Experimental groups, see materials and methods; Section 2.8. RBG, random blood glucose; FBG, fasting blood glucose level measured in blood serum of 12h-fasted rats. ^a–d^ No significant difference (*p* > 0.05) between any two means within the same column that have the same superscripted letters. ^A–C^ No significant difference (*p* > 0.05) between any two means with the same superscripted letters within the same row.

**Table 5:** Effect of orally administrated FOE and HFOE on lipid profile (mg dL^−1^) and atherogenic index in STZ-induced diabetes in rats (mean ± SE), *n* = 8.

Groups *	Lipid Profile Parameters					
	TG	CHO	HDL-CHO	LDL-CHO	VLDL-CHO	AI
NR	72.88 ± 3.43 ^c^	89.73 ± 9.99 ^b^	33.33 ± 2.28 ^a^	43.49 ± 12.37 ^cd^	14.58 ± 0.69 ^c^	0.37 ± 0.09 ^c^
DR	119.07 ± 3.82 ^a^	138.61 ± 10.34 ^a^	26.52 ± 2.43 ^b^	88.28 ± 13.70 ^a^	23.81 ± 0.77 ^a^	0.70 ± 0.10 ^a^
DR + MET	101.74 ± 11.16 ^ab^	107.58 ± 12.63 ^b^	36.36 ± 4.55 ^a^	55.41 ± 2.55 ^bc^	20.35 ± 2.23 ^a^	0.45 ± 0.08 ^b^
DR + FOE1	92.29 ± 6.05 ^b^	111.2 ± 7.10 ^ab^	28.79 ± 2.20 ^b^	67.74 ± 6.77 ^b^	18.46 ± 1.21 ^b^	0.54 ± 0.7 ^b^
DR + FOE2	84.33 ± 4.93 ^bc^	107.84 ± 13.39 ^b^	38.64 ± 3.08 ^a^	58.98 ± 7.48 ^bc^	20.35 ± 2.23 ^ab^	0.36 ± 0.05 ^c^
DR + HFOE1	99.25 ± 7.96 ^b^	104.99 ± 3.44 ^b^	34.09 ± 4.02 ^a^	54.50 ± 4.48 ^bc^	19.85 ± 1.59 ^ab^	0.47 ± 0.07 ^b^
DR + HFOE2	87.07 ± 3.73 ^bc^	99.82 ± 11.96 ^b^	37.88 ± 1.92 ^a^	44.53 ± 7.70 ^cd^	17.81 ± 0.57 ^bc^	0.35 ± 0.02 ^c^

* Experimental groups, see materials and methods; Section 2.8. TG, triglycerides; CHO, total cholesterol; HDL-CHO, high-density-lipoprotein-cholesterols; LDL-CHO, low-density-lipoprotein-cholesterols; VLDL-CHO, very-low-density-lipoprotein-cholesterols; AI, atherogenic index. ^a–d^: There is no significant difference (*p* > 0.05) between any two means within the same column that have the same superscripted letters.

**Table 6:** Effect of orally administrated FOE and HFOE on liver function biomarkers in STZ-induced diabetes in rats (mean ± SE), *n* = 8.

Groups *	Liver Function Biomarkers			
	ALT (U L^−1^)	AST(U L^−1^)	ALP(U L^−1^)	T. Bili (mg dL^−^^1^)
NR	44.50 ± 3.46 ^b^	57.66 ± 5.37 ^b^	60.88 ± 12.33 ^c^	0.69 ± 0.2 ^c^
DR	34.50 ± 3.46 ^c^	87.66 ± 9.26 ^a^	157.38 ± 7.41 ^a^	1.06 ± 0.23 ^a^
DR + MET	46.43 ± 2.12 ^b^	68.84 ± 7.50 ^ab^	105.11 ± 11.60 ^b^	0.75 ± 0.10 ^b^
DR + FOE1	57.19 ± 1.96 ^a^	68.84 ± 7.50 ^ab^	97.76 ± 11.37 ^b^	0.75 ± 0.05 ^b^
DR + FOE2	49.84 ± 4.75 ^b^	69.90 ± 2.01 ^ab^	76.85 ± 9.46 ^c^	0.66 ± 0.09 ^c^
DR + HFOE1	54.29 ± 2.78 ^a^	76.49 ± 5.95 ^ab^	91.72 ± 13.51 ^b^	0.73 ± 0.06 ^b^
DR + HFOE2	47.62 ± 3.05 ^b^	64.78 ± 8.28 ^b^	77.08 ± 5.94 ^c^	0.67 ± 0.08 ^c^

* Experimental groups, see materials and methods; Section 2.8. ALT, alanine aminotransferase; AST, aspartate aminotransferase; ALP, alkaline phosphatase; T. Bili, total bilirubin. ^a–c^ No significant difference (*p* > 0.05) between any two means within the same column that have the same superscripted letters.

**Table 7:** Effect of orally administrated FOE and HFOE on kidney function biomarkers in STZ-induced diabetes in rats (mean ± SE), *n* = 8.

Group *	Kidney Function Biomarkers					
	T. Protein (g dL^−1^)	Albumin (g dL^−1^)	Globulin (g dL^−1^)	Creatinine (mg dL^−1^)	Urea (mg dL^−1^)	BUN (mg dL^−1^)
NR	8.91 ± 0.19 ^b^	4.14 ± 0.29 ^a^	4.77 ± 0.23 ^a^	0.79 ± 0.04 ^b^	31.44 ± 4.90 ^b^	14.78 ± 4.08 ^b^
DR	6.96 ± 0.32 ^d^	3.58 ± 0.09 ^b^	3.37 ± 0.32 ^b^	1.29 ± 0.05 ^a^	74.63 ± 3.62 ^a^	35.08 ± 6.26 ^a^
DR + MET	7.57 ± 0.36 ^cd^	4.08 ± 0.40 ^a^	3.49 ± 0.61 ^ab^	0.95 ± 0.09 ^b^	44.58 ± 5.88 ^b^	20.95 ± 2.76 ^b^
DR + FOE1	7.31 ± 0.28 ^d^	3.66 ± 0.26 ^ab^	3.66 ± 0.45 ^ab^	0.91 ± 0.09 ^b^	44.58 ± 5.88 ^b^	20.11 ± 1.49 ^b^
DR + FOE2	7.96 ± 0.48 ^cd^	3.86 ± 0.38 ^ab^	4.11 ± 0.56 ^a^	0.83 ± 0.07 ^b^	44.43 ± 7.35 ^b^	20.88 ± 3.45 ^b^
DR + HFOE1	8.40 ± 0.33 ^bc^	3.55 ± 0.29 ^ab^	4.85 ± 0.47 ^a^	0.97 ± 0.12 ^b^	46.92 ± 4.29 ^b^	21.21 ± 5.98 ^b^
DR + HFOE2	10.25 ± 0.34 ^a^	4.26 ± 0.22 ^a^	4.66 ± 0.42 ^a^	0.82 ± 0.05 ^b^	38.21 ± 7.46 ^b^	17.96 ± 3.50 ^b^

* Experimental groups, see materials and methods; Section 2.8. ^a–d^: No significant difference (*p* > 0.05) between any two means within the same column that have the same superscripted letters.

## Data Availability

Data is contained within the article.

## Abbreviations

ABTS, 2,2′-azino-bis (3-ethylbenzothiazoline-6-sulfonic acid); ALT, Alanine aminotransferase; AST, Aspartate aminotransferase; CAT, Catalase; CHO, Total cholesterol; DPPH: 1,1-diphenyl-2-picryl hydrazine; DW: Dry weight; FBG, Fasting blood glucose; FOE, Fermented oat; GA: Gallic acid; GAE: Gallic acid equivalent; GSH: Reduced-glutathione; HDL, High-density lipoproteins; HFOE, honey Fermented oat; LDL, Low-density lipoproteins; MDA: Malonaldehyde; RSA: Radical scavenging activity; SE: Standard error; SOD: Superoxide dismutase; TBA, Thiobarbituric acid; TE: Trolox equivalents; TG, Triglycerides; TPC, Total phenolic content; VLDL, Very-Low-density lipoproteins.
